# LIF promotes neurogenesis and maintains neural precursors in cell populations derived from spiral ganglion stem cells

**DOI:** 10.1186/1471-213X-7-112

**Published:** 2007-10-12

**Authors:** Kazuo Oshima, Dawn Tju Wei Teo, Pascal Senn, Veronika Starlinger, Stefan Heller

**Affiliations:** 1 Stanford University School of Medicine, Departments of Otolaryngology, Head & Neck Surgery and Molecular & Cellular Physiology, Stanford CA, USA; 2 Department of Otolaryngology Head & Neck Surgery, Singapore General Hospital, Singapore Health Services, Singapore; 3 Department of Otolaryngology, Head & Neck Surgery, Inselspital, University of Berne, Switzerland

## Abstract

**Background:**

Stem cells with the ability to form clonal floating colonies (spheres) were recently isolated from the neonatal murine spiral ganglion. To further examine the features of inner ear-derived neural stem cells and their derivatives, we investigated the effects of leukemia inhibitory factor (LIF), a neurokine that has been shown to promote self-renewal of other neural stem cells and to affect neural and glial cell differentiation.

**Results:**

LIF-treatment led to a dose-dependent increase of the number of neurons and glial cells in cultures of sphere-derived cells. Based on the detection of developmental and progenitor cell markers that are maintained in LIF-treated cultures and the increase of cycling nestin-positive progenitors, we propose that LIF maintains a pool of neural progenitor cells. We further provide evidence that LIF increases the number of nestin-positive progenitor cells directly in a cell cycle-independent fashion, which we interpret as an acceleration of neurogenesis in sphere-derived progenitors. This effect is further enhanced by an anti-apoptotic action of LIF. Finally, LIF and the neurotrophins BDNF and NT3 additively promote survival of stem cell-derived neurons.

**Conclusion:**

Our results implicate LIF as a powerful tool to control neural differentiation and maintenance of stem cell-derived murine spiral ganglion neuron precursors. This finding could be relevant in cell replacement studies with animal models featuring spiral ganglion neuron degeneration. The additive effect of the combination of LIF and BDNF/NT3 on stem cell-derived neuronal survival is similar to their effect on primary spiral ganglion neurons, which puts forward spiral ganglion-derived neurospheres as an *in vitro *model system to study aspects of auditory neuron development.

## Background

Sphere-forming stem cells of the inner ear, characterized by their ability to self-renew and to differentiate into multiple cell types, can be isolated from vestibular sensory epithelia as well as from the cochlear organ of Corti and the spiral ganglion [1-10]. Spiral ganglion-derived spheres have been first isolated from the adult guinea pig and human inner ear [3] and, more recently, from murine inner ear tissue [7,8]. These spheres consist of a mixed population of a few *bona fide *stem cells, neural progenitor cells, and differentiating cell types; they can be distinguished from sensory epithelia-derived spheres by their ease of dissociation and by their grape cluster-like morphology [3,7,8]. Inner ear-derived stem cells have been put forward as a potential source of replacement cells for sensory hair cell and auditory neuron degeneration, which are the leading causes of hearing impairment, affecting more than 250 million people worldwide [11-13].

LIF is well known for promoting self-renewal of murine embryonic [14] as well as murine and human neural stem cells [15,16]. It binds to a heterodimeric membrane receptor complex consisting of LIF receptor (LIFR, also described as LIFR-beta) and glycoprotein 130 (gp130), which leads to the activation of the Janus kinase – signal transducer and activator of transcription (Jak-STAT) pathway [17,18]. Other known cytokines signaling via the same principal pathway are ciliary neurotrophic factor, interleukin-6, interleukin-11, oncostatinM, cardiotrophin-1, and cardiotrophin-like cytokine [19]. The effects of LIF and related cytokines on distinct progenitor cell populations in development and in response to injury are promiscuous. They range from modulation of neurogenesis [20], to control of glial responses to injury [21], and to affecting neural progenitors *in vitro *[15,16] and *in vivo *[22,23]. In the inner ear, LIF has been shown to promote the survival of spiral ganglion neurons in culture, acting synergistically with the neurotrophins BDNF and NT3 [24,25].

In this study, we investigated whether treatment with LIF increases the self-renewal capacity of spiral ganglion stem cells. We found that treatment with LIF affected the adherence properties of spiral ganglion spheres, making it impossible to propagate spiral ganglion stem cells as spheres. However, we encountered a strong dose-dependent effect of LIF on neural cell differentiation. This effect is based on several mechanisms including increased proliferation and decreased apoptosis of neural progenitors, but most prominently by a direct promotion of neural differentiation. Overall, our results show that LIF and neurotrophins strongly promote neurogenesis of progenitors derived from spiral ganglion stem cells, and that LIF alone is capable of maintaining a pool of cycling neural progenitors derived from spiral ganglion stem cells.

## Results

### LIF treatment causes floating spiral ganglion-derived spheres to adhere and inhibits primary sphere formation

We set out to test whether LIF affects self-renewal of spiral ganglion stem cells by culturing spheres in the presence of LIF. Within 45 minutes after addition of LIF to floating spheres, all spheres started to attach to the plastic bottoms of the suspension culture dishes used for maintenance of floating spheres. After 3 hours, 100% of the spheres were attached at all concentrations tested (from 0.1 – 10 ng, n = 10). Spheres did not attach when we added 0.002% BSA (vehicle control) or other unrelated recombinant factors (BDNF or NT3) from the same supplier. This effect made it unfeasible to culture and to propagate spheres in the presence of LIF, which made it impossible to determine potential effects of LIF on the self-renewal of spiral ganglion-derived sphere-forming stem cells under non-adherent conditions. Furthermore, we noted that adding LIF at a concentration of 10 ng/ml completely inhibited formation of primary spheres from dissociated spiral ganglion cells. These effects of LIF are not without precedent as it has been reported that LIF impairs the formation of neurospheres from embryonic brain-derived neural stem cells and that it leads to attachment of neurospheres [26].

### Increase of TuJ- and GFAP-positive cells in LIF-treated sphere-derived cultures

**Figure 1:**
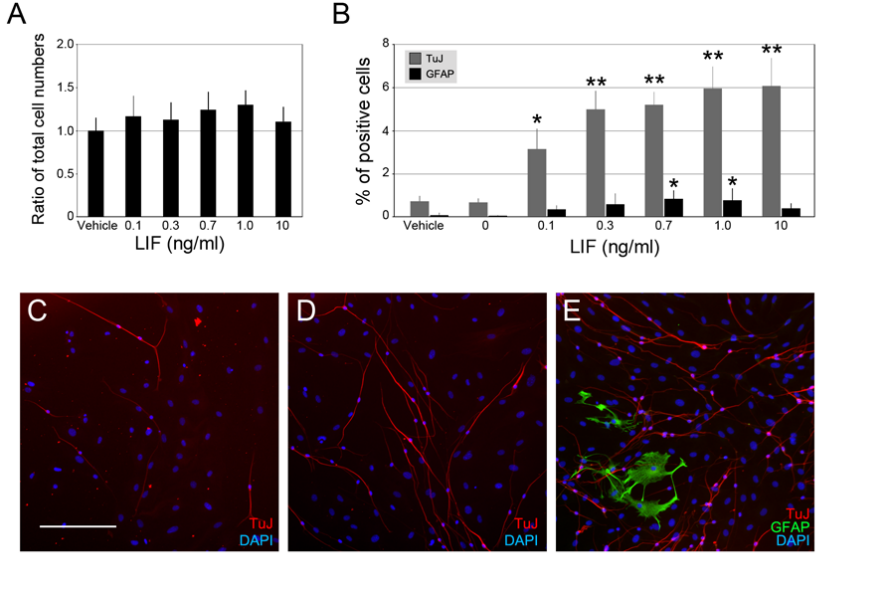
LIF treatment leads to an increase of TuJ and GFAP positive cells without affecting the total cell number. (**A**): The total cell number of sphere-derived cells after 10 days in culture did not significantly change when treated with different concentrations of LIF. *n *= 4. (**B**): The percentage of TuJ-positive cells was significantly increased in a dose-dependent manner up-to 7-fold. Likewise, the percentage of GFAP-expressing cells was increased in response to LIF. The percent values express the fraction of immunopositive cells of the total cell number. Error bars indicate S.E.M. * indicates *p *< 0.05, ** indicates *p *< 0.01, *n *= 6. (**C-E**): Representative images of differentiated cells of non-LIF-treated cultures (C), and cultures treated with 0.1 ng/ml (D) and 10 ng/ml LIF (E). Scale bar = 200 μm.

To investigate a potential effect of LIF on sphere-derived adherent cell populations, we cultured the attached cells from 30 spheres for each experiment in differentiation medium (defined in the Methods section below) in the presence or absence of LIF. After 10 days, we determined the total number of cells, the number of TuJ-positive cells with neural morphology, and the number of cells expressing the glial marker GFAP. We noticed that the total cell number did not significantly change when we compared cultures treated with different concentrations of LIF to control cultures (Fig. 1A). The number of TuJ-positive cells, however, was significantly increased in a dose-dependent manner reaching a maximum at 1 ng/ml LIF, with no further increase even with a 10x higher concentration of LIF (Fig. 1B–E). Only a few GFAP-positive cells were detectable in sphere-derived cell populations cultured in the absence of LIF, but with increasing LIF concentration, we observed higher numbers of GFAP-expressing cells. The number of GFAP-positive cells was significantly increased when compared with controls at 0.7 ng/ml and 1 ng/ml LIF (Fig. 1B). To verify that the neurons and glial cell types differentiated during the 10-day culture period, we analyzed sphere cell populations 3 hours after attachment and did not detect TuJ- or GFAP-positive cells (data not shown), demonstrating that at the onset of the experiment, spheres do not contain differentiated neurons and glial cells.

### LIF maintains neural progenitor cells in sphere-derived cultures

**Figure 2:**
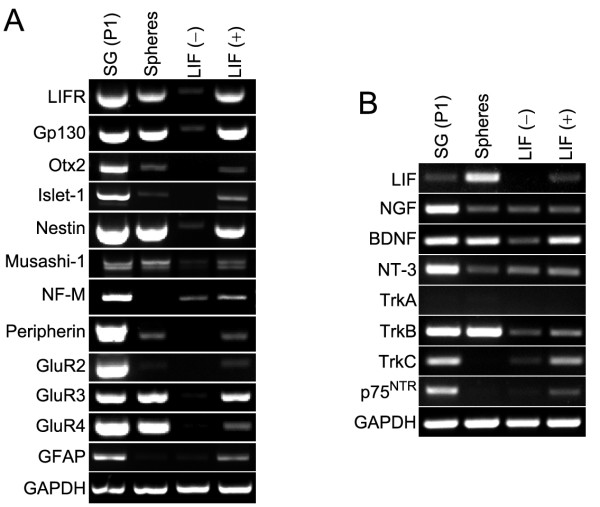
(**A-B**): RT-PCR analyses of marker gene expression in spiral ganglion from P1 mouse (SG(P1), first column of lanes), spiral ganglion-derived spheres (second column of lanes), differentiated spheres without LIF treatment after a 10-day culture period (LIF(-), third column of lanes), and differentiated spheres treated with 10 ng/ml of LIF after a 10-day culture period (LIF(+), fourth column of lanes). None of the two TrkA primer pairs employed in this study amplified TrkA cDNA from RNA preparations of spiral ganglia, spheres, or sphere-derived cells – one representative result is shown. Both primer pairs were successfully tested in control experiments with mouse dorsal root ganglia and hindbrain cDNA as template (not shown).

Using RT-PCR, we analyzed the expression of mRNA encoding the receptor components of the LIF signaling pathway and found that LIFR and gp130 were robustly expressed in spheres and also in sphere-derived cell populations cultured for 10 days in differentiation medium in the presence of 10 ng/ml LIF (Fig. 2A). Both genes were expressed weaker in cultures that were not treated with LIF. LIFR and gp130 were also detectable in spiral ganglia, validating the previously reported effects of LIF on spiral ganglion neuron survival [24,25]. LIF mRNA expression was detectable in spiral ganglia and robustly in spheres (Fig. 2B). After 10 days culture of sphere-derived cells without LIF addition, we did not detect LIF expression; when LIF protein was added, however, LIF mRNA was unambiguously detectable.

Otx2 is a transcription factor that appears to have multiple roles in inner ear development including a proposed involvement in the specification of the spiral ganglion [27-29]. We detected Otx2 mRNA in the neonatal spiral ganglion, as well as in spiral ganglion-derived spheres (Fig. 2A). Interestingly, LIF-treatment led to robust maintenance of this developmentally important transcription factor, whereas Otx2 expression was not detectable in untreated cultures. A similar expression profile was observed for islet-1, a gene expressed in early auditory and vestibular neurons [30]. The two neural progenitor markers nestin [31] and musashi-1 [32] were also robustly expressed in the neonatal spiral ganglion and spiral ganglion-derived spheres and were maintained in sphere-derived LIF-treated cultures (Fig. 2A). The general expression pattern observed with developmental and progenitor cell genes suggests that LIF appears to sustain a pool of inner ear neural progenitor cells in cultures derived from spiral ganglion spheres. This pool of cells seems to lose expression of developmental and precursor markers in the absence of LIF. RT-PCR analysis also confirmed our immunocytological results suggesting upregulation and/or maintenance of the neural markers neurofilament-M, peripherin, GluR2, GluR3, and GluR4 [33,34], as well as the glial marker GFAP in LIF-treated sphere-derived cultures (Fig. 2A).

Expression of NGF, BDNF, and NT-3 was strong in spiral ganglia (Fig. 2B). BDNF appeared to be expressed at relative high levels in spheres and after LIF-treatment, whereas the other two neurotrophins were maintained at lower levels, compared with BDNF expression. Expression of TrkA mRNA was not detectable in any of the cell populations analyzed, whereas TrkB was robustly expressed in spiral ganglia and spheres, and moderately in sphere-derived cultures. TrkC was detectable in spiral ganglia, absent in spheres, detectable at low levels in non-LIF-treated cultures, and appeared to be upregulated in response to LIF. The low-affinity neurotrophin receptor p75^NTR ^was expressed in spiral ganglia and in sphere-derived cell cultures treated with LIF.

### Increased BrdU incorporation and increased number of neural progenitor cells in LIF-treated cultures

**Figure 3:**
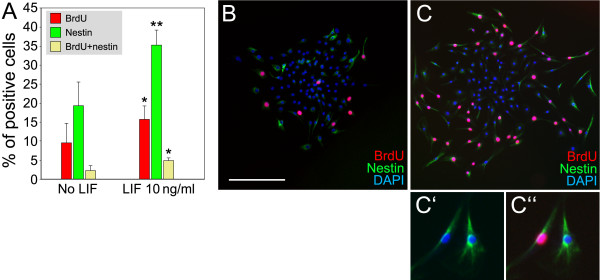
Increased BrdU incorporation and increased number of neural progenitor cells in response to LIF treatment. (**A**): Quantification of BrdU-positive, nestin-positive, and BrdU/nestin-double positive cells 48 h after plating. Error bars indicate S.E.M., * indicates *p *< 0.05, ** indicates *p *< 0.01, *n *= 8. Total cell numbers did not differ significantly when we compared LIF-treated with untreated cultures (1934 ± 403 and 2008 ± 435, respectively). (**B-C**): Representative pictures of plated sphere-derived cells without LIF treatment (B), and with 10 ng/ml LIF (C). Scale bar = 200 μm. (**C'-C''**): Higher magnification of two nestin-positive cells from (C) to illustrate the nuclear localization of the BrdU staining in one of the cells.

To further elucidate the mechanism(s) by which LIF affects spiral ganglion stem cell-derived progenitor populations, we determined whether LIF modulates the number of proliferating cells. In the first 48 hours of culturing attached spheres in differentiation medium in the presence of LIF, we found that the number of cells that incorporated the thymidine analog BrdU was significantly increased by 64% (Fig. 3A). Furthermore, LIF-treatment led to a twofold increase of nestin-positive cells as well as a doubling of the BrdU and nestin double-positive cell population (Fig. 3A–C). This experiment revealed two different effects of LIF. First, LIF appears to promote cell proliferation of nestin-negative as well as of nestin-positive cells, which in part explains the overall increase of nestin-positive cells. Nevertheless, the 64% increase of total BrdU-positive cells cannot completely account for the stark expansion of the nestin-positive cell population. Consequently, we propose a second effect of LIF, which is induction of nestin expression in nestin-negative sphere-derived cells.

### LIF promotes differentiation of neural progenitors

**Figure 4:**
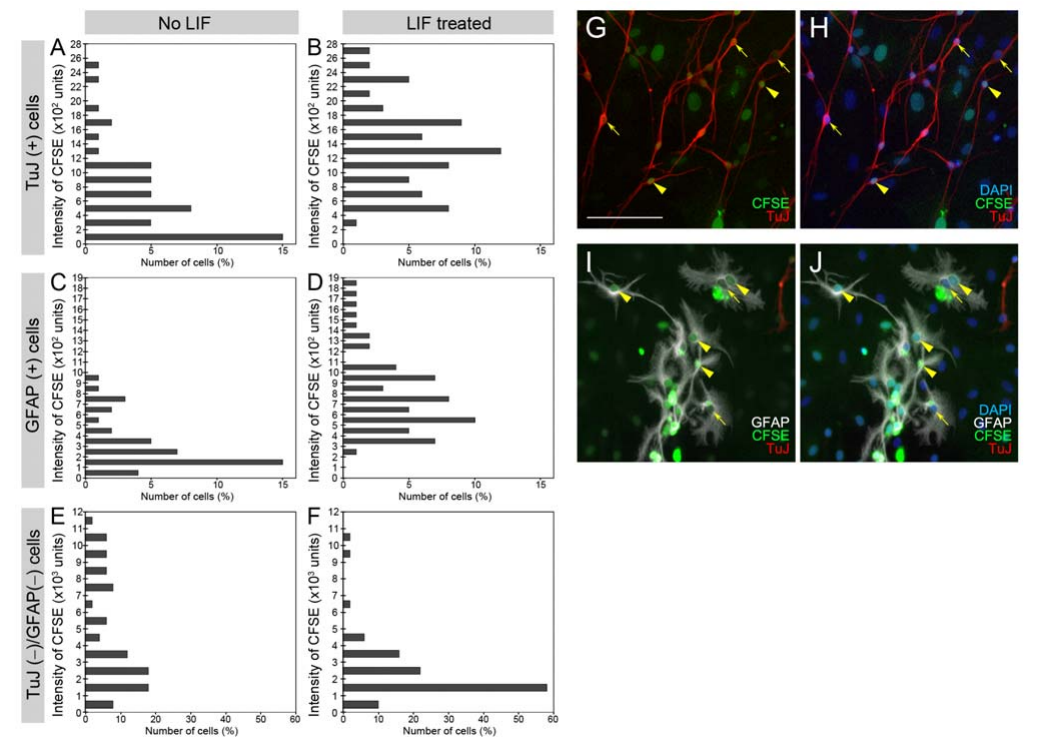
Analysis of cell divisions of TuJ and GFAP expressing cells, and TuJ and GFAP-negative cells in response to LIF. (**A-F**): CFSE intensity distribution histograms comparing control cultures (no LIF) with LIF-treated cultures for TuJ-positive (A,B), GFAP-positive (C,D), and TuJ & GFAP-negative (E,F) cells. The distribution shifts indicate that TuJ and GFAP-expressing cells retained more CFSE label in LIF-treated cultures when compared with untreated cultures. The TuJ & GFAP-negative cells retained less label, suggesting that these cells underwent more divisions in response to LIF. (**G-H**): CFSE signals (green) in TuJ-positive cells (red). Nuclei are shown in blue. (**I-J**): CFSE signals (green) in GFAP-positive cells (white). Nuclei are shown in blue. Arrowheads point to cells displaying well retained CFSE label and arrows indicate cells with less intense CFSE label. Scale bar = 100 μm.

We hypothesized that LIF could also be directly affecting neural differentiation of progenitor cells, thereby, decreasing the mitotic potency of neural progenitors. As BrdU showed detrimental effects when we cultured spiral ganglion sphere-derived cells for longer than 4 days, we used an alternative method for tracing proliferating cells. Three hours after attachment, sphere-derived cells were loaded with carboxy fluorescein diacetate succinimidyl ester (CFSE) and then cultured for 12 days in differentiation medium in the presence or absence of LIF. Mitotic cell divisions distribute the fluorescent CFSE compound between the daughter cells, which results in a decrease of the fluorescence intensity with each round of cell division. Analysis of CFSE fluorescence intensity after 12 days revealed that the majority of TuJ- and GFAP-positive cells in LIF-treated cultures retained the CFSE label at higher levels than in untreated cultures (Fig. 4A–D,G–J). This result indicates that the neurons and glial cells in LIF-treated cultures arose from progenitors that underwent fewer mitotic division cycles than those in untreated cultures, which suggests that LIF directly induced neural and glial cell differentiation. Together with the observation that LIF directly promotes the generation of nestin-positive cells (Fig. 3A), we conclude that LIF has the ability to accelerate differentiation of neural progenitors.

We also analyzed the distribution of CFSE in TuJ- and GFAP-negative cells and found that LIF affected these cells in an opposite manner, manifesting in a lower concentration of retained CFSE label when compared with untreated controls (Fig. 4E,F). This result is in agreement with our observation that the overall cell number does not significantly change compared to untreated controls (Fig. 1A).

### LIF broadly promotes survival in sphere-derived cultures

**Figure 5:**
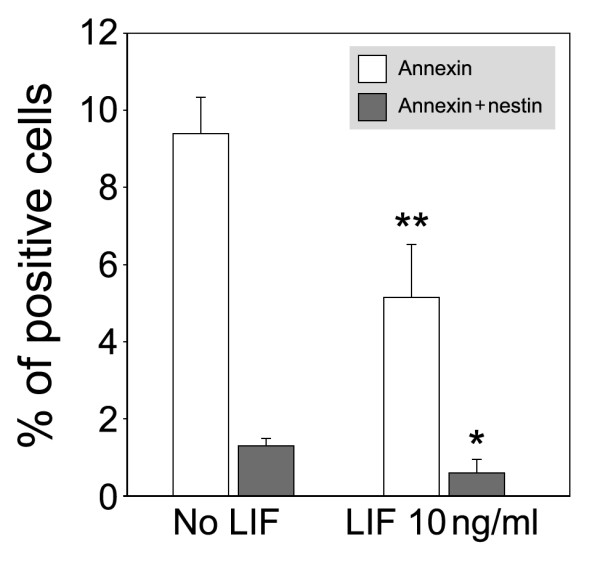
Reduction of apoptotic cells in LIF treated sphere-derived cell cultures 48 h after plating. Annexin-positive cells and annexin & nestin-double positive cell populations were significantly decreased in response to LIF. Error bars indicate S.E.M., * indicates *p *< 0.05, ** indicates *p *< 0.01, *n *= 4. Total cell numbers did not differ significantly when we compared LIF-treated with untreated cultures (1124 ± 363 and 894 ± 516, respectively).

An additional mechanism by which LIF could increase the number of neural and glial cells in spiral ganglion stem cell-derived cell populations is by promoting cell survival. We used AnnexinV to detect phosphatidylserine, which is redistributed in apoptotic cells from the internal to the external plasma membrane leaflet [35]. In the first 48 hours of culturing attached spheres in differentiation medium, we found that in the presence of LIF, the number of apoptotic cells was significantly reduced (Fig. 5). The number of nestin-positive apoptotic cells was equally reduced, indicating that LIF appears to generally promote cell survival in spiral ganglion sphere-derived cell populations.

### LIF, BDNF, and NT3 act in concert to promote neural differentiation and survival

**Figure 6:**
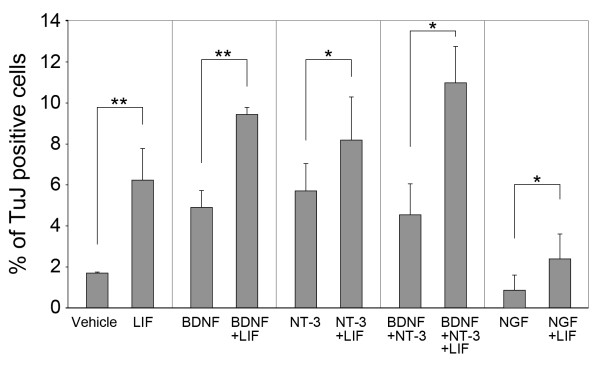
Additive effects of LIF, BDNF, and NT-3 on neurogenesis. Quantification of the fraction of TuJ-expressing cells of the total number of cells treated with LIF and different combinations of neurotrophins. The analysis was done after a 10-day differentiation period. LIF was used at 10 ng/ml, BDNF and NT-3 at 50 ng/ml, and NGF at 20 ng/ml. Error bars indicate S.E.M., * indicates *p *< 0.05, ** indicates *p *< 0.01, *n *= 3.

It has previously been reported that LIF and the neurotrophins BDNF and NT3 act synergistically in survival assays of neonatal primary spiral ganglion neurons [24]. Although we did not observe a synergistic action on neuron numbers in spiral ganglion sphere-derived cell populations, we found an additive effect (Fig. 6). Co-treatment with LIF, BDNF, and NT3 led to the highest fraction of neurons, up-to a maximum of 15% of the total cell numbers. NGF, as expected for inner ear-derived neural populations [36], was not effective. In combination with LIF, however, NGF-treatment resulted in fewer TuJ-expressing cells than in the appropriate controls indicating that NGF may exert a negative effect on neurogenesis in these conditions. Nevertheless, the observed effects of neurotrophins BDNF, NT3, and LIF on sphere-derived neuron survival are very similar to the effects of these factors on primary spiral ganglion neurons isolated from newborn mice [25].

## Discussion

Spiral ganglion-derived stem cells are promising candidates for generating replacement auditory neurons, which could be used in transplantation experiments with animal models of auditory nerve degeneration [37,38]. In this study, we focused on determining possible functions of the cytokine LIF on progenitor cells that reside in spheres generated from spiral ganglion stem cells. We found that LIF strongly impairs the formation and propagation of spiral ganglion-derived spheres. A qualitatively similar effect of LIF has been reported on fetal neural stem cells [26]. We consequently focused on the potential roles of LIF on sphere-derived adherent cell populations, and revealed several mechanisms by which LIF promotes the formation of neurons and GFAP-positive glial cells.

Our results differ quantitatively from a previous study of sphere-derived cells that were isolated from adult guinea pigs [3]. In this previous study, most cells expressed glial or neural markers, whereas we found that most sphere-derived cells did neither express GFAP or TuJ. We hypothesize that these distinctions could be due to differences in experimental design, which includes use of different species, use of different immunological tools, different time points of analyses, and different combinations of added factors.

We demonstrate that LIF treatment in a dose-dependent manner increases the number of neurons and glial cells that differentiated in cultures of sphere-derived cells. These neurons and glial cells were newly generated because neither cell type was detectable when we analyzed spheres directly after plating. By RT-PCR, we revealed that developmental and progenitor cell markers are maintained in LIF-treated cultures, whereas their expression was starkly reduced or absent in untreated cultures. These results suggest that LIF has a function in maintaining neural progenitor cells in spiral ganglion sphere-derived cultures.

LIF mRNA was detectable at relatively low levels in P1 spiral ganglia, but was robustly found in spheres and was maintained in sphere-derived cultures in the presence of LIF. It is noteworthy that addition of LIF to spiral ganglion cells inhibited sphere formation, but on the other hand, LIF mRNA expression was detectable in spheres. This indicates that LIF might indeed play a role in stem cell or progenitor cell maintenance in spheres.

When we cultured sphere-derived cell populations, we found that LIF-treatment resulted in an increase of cells that incorporated BrdU. This growth-promoting effect was detectable in both nestin-positive and nestin-negative cells. Nestin-positive cells are neural progenitor cells, and the effect of LIF on those cells may account for the maintenance of a pool of cycling nestin-expressing progenitors in LIF-treated cultures. We further noted that the overall fraction of nestin-positive cells was starkly increased in response to LIF; this increase was disproportionately large compared to the effect of LIF on proliferating nestin-positive progenitors. We consequently conclude that LIF affected a population of nestin-negative progenitor cells to develop into neural progenitors. Our results do not directly address the question of whether these LIF-induced nestin-positive cells are proliferating. Our CFSE label-retention experiments suggest, however, that in the presence of LIF, many neural and glial precursors directly differentiate into mature cell types whereas in untreated cultures, the precursors proliferated more strongly.

In summary, we propose that LIF affects distinct populations of progenitor cells in spiral ganglion sphere-derived cell populations in different manners. First, LIF promotes the maintenance of cycling nestin-positive progenitor cells. These cells may be the pool of stem cells that originally had the ability to form spheres; a hypothesis that needs to be further tested. Second, LIF increases the number of nestin-positive progenitor cells in a cell cycle-independent fashion, probably by inducing neural progenitor cell features in a population of nestin-negative cells. Third, LIF accelerates neurogenesis in the majority of neural progenitors by promoting their differentiation at the cost of their proliferative potential. This proposed third role of LIF somewhat contradicts the action of maintaining circling progenitors. A possible explanation for such an antidromic effect is the heterogeneity of sphere-derived cell populations, where different sub pools of progenitors appear to be affected in a different manner. We hypothesize that all LIF actions are in agreement with a general role in promoting neurogenesis and simultaneous maintenance of a precursor cell pool, which puts this cytokine forward as a powerful tool to control the fate of spiral ganglion stem cell-derived progenitor populations *in vitro*.

The multiple effects of LIF on spiral ganglion sphere-derived cells became even more obvious when we compared the number of apoptotic cells in treated and untreated populations. Here, we noticed a general ability of LIF to promote survival of both nestin-positive and nestin-negative cells. Anti-apoptotic action of LIF has been previously observed [39,40], and this effect seems to contribute to the overall health and robust neurogenesis that we observed in LIF-treated cultures of spiral ganglion sphere-derived cells.

Simultaneous treatment with LIF and the neurotrophins BDNF and NT3 resulted in an increased occurrence of neurons when compared with cultures treated with each factor alone. We interpret this additive effect as the result of independent and overlapping neurogenic and trophic mechanisms. These include the neurogenic actions of LIF on progenitors, the trophic effects of LIF on sphere-derived cells, as well as the neurotrophic effects of BDNF and NT3 on neurons. Auditory and vestibular neurons depend on BDNF and NT3, but not on NGF [36]. NGF alone had no effect on the number of neurons generated in sphere-derived cultures, but in conjunction with LIF, we observed a decrease in the number of differentiated neurons.

Neurotrophins act via binding to the high-affinity Trks and the low-affinity neurotrophin receptor p75^NTR^. Only TrkB and TrkC mRNAs were detectable in sphere-derived cultures, which is in agreement with previous studies of sphere-derived cell populations made from guinea pig spiral ganglion [3]. TrkB and BDNF mRNAs were robustly detectable in spheres, indicating that this signaling pathway might be distinctly active in these cell populations, when compared with the other two neurotrophins. We did not detect TrkA mRNA in spiral ganglia, in spiral ganglia-derived spheres, and in sphere-derived cultures, which is in agreement with the majority of previous studies ([41-44], but see [45]). We consequently hypothesize that the reduction of the fraction of TuJ-positive cells in response to NGF in LIF-treated cultures (Fig. 6) could be a result of NGF action on p75^NTR^. Expression of the low-affinity neurotrophin receptor p75^NTR ^was indeed detectable in spiral ganglia and in sphere-derived cell populations after treatment with LIF (Fig. 2B). p75^NTR ^interacts with Trks resulting in enhancement of their ligand specificity and affinity, thereby modulating Trk-mediated neuronal survival (reviewed in [46]). Other studies have revealed that activation of p75^NTR ^can also mediate apoptosis in a Trk-independent fashion (reviewed in [47]). We speculate that the negative effect of NGF on neurogenesis in cultures that were simultaneously treated with LIF could be the result of an apoptotic effect via p75^NTR ^signaling.

The expression of peripherin and glutamate receptor subtypes in sphere-derived cells, particularly after LIF treatment indicates that sphere-derived populations maintain a distinct spiral ganglion neuron phenotype. Such maintenance of organ-specific cellular identity, a feature of many somatic stem cell-derived cell populations, qualifies spiral ganglion-derived sphere-forming stem cells as a *bona fide *cell source for transplantation studies toward replacement of lost peripheral auditory neurons.

## Conclusion

We demonstrate that LIF is highly efficient in promoting neurogenesis of spiral ganglion stem cell-derived cultures. We propose that LIF treatment may be useful to expand adherent spiral ganglion stem cell populations and, furthermore, that such a treatment may enhance the number of auditory neurons after transplantation of LIF-treated spiral ganglion sphere-derived cells.

## Methods

### Isolation of spiral ganglion stem cells for sphere formation

Postnatal day 1 (P1) C57/BL6 mice were used for each experiment. All procedures followed the approved institutional protocol according to the National Institutes of Health guidelines for animal care. The otic capsule was isolated and dissected carefully from the surrounding tissue. The capsule was then opened apically and removed with forceps followed by dissecting away the spiral ligament and stria vascularis. The organ of Corti was peeled off from the modiolus, which harbors spiral ganglion cells and neuronal fibers. The dissected spiral ganglia were washed with ice-cold Hanks' media. Individual ganglia were then transferred into 50 μl drops of PBS. 50 μl of 0.25% trypsin solution with EDTA (#25200-056, Invitrogen) was added and the tissue was incubated for 5 minutes at 37°C. The enzymatic reaction was blocked with 100 μl of a solution consisting of 10 mg/ml soybean trypsin inhibitor (Worthington) and 1 mg/ml DNaseI (Worthington) in DMEM/high-glucose and F12 media (mixed 1:1). Gentle trituration was performed with plastic pipette tips (epTIPS Filter 20–300 μl, Eppendorf). The cell suspension was further diluted with 1.8 ml of DMEM/high-glucose and F12 media (mixed 1:1, Invitrogen) supplemented with N2 and B27 supplements, EGF (20 ng/ml), bFGF (10 ng/ml), IGF-1 (50 ng/ml), and heparan sulphate (50 ng/ml) (all growth factors were obtained from R&D Systems and Sigma). Larger tissue fragments and cellular debris were removed by passing the cell suspension through a 70 μm cell strainer directly into a 6-well cell suspension culture plate (Greiner Bio-One). This procedure generally yielded a completely dissociated single cell suspension virtually devoid of aggregates. The cells were incubated at 37°C for 7 days to allow for primary sphere formation. Primary spheres were passaged after 7 days in culture by a 7 minute treatment with Accumax (Innovative Cell Technologies) at 37°C, followed by mild 5 minute centrifugation (150rcf). The supernatant was aspirated leaving about 200 μl. Using a fire polished glass pipette, the pellet was triturated and the resulting cell suspension was resuspended in 1.8 ml of DMEM/high-glucose and F12 media (mixed 1:1) supplemented with N2 and B27 supplements, EGF (20 ng/ml), bFGF (10 ng/ml), IGF-1 (50 ng/ml), and heparan sulphate (50 ng/ml). We microscopically ensured that a single cell suspension was generated. This suspension was then incubated at 37°C for 7 days to obtain second-generation spheres. We have previously shown that this procedure is feasible for long-term propagation of spiral ganglion-derived spheres [7,8].

### Cell differentiation

To study cell differentiation, 30 equally sized second-generation spheres were transferred per experimental data point into plastic 4-well tissue culture plates (Greiner 35/10 mm 4-well tissue culture dishes) coated with 0.1% gelatin (Chemicon). Recombinant rat LIF (Chemicon, #LIF3005) was added directly into the differentiation medium consisting of DMEM/high-glucose and F12 media (mixed 1:1) supplemented with N2 at 37°C. 80% of the medium was replaced every other day. The differentiated cells were analyzed by immunocytochemistry after 10 days to assess the dose-dependent characteristics of LIF-treatment. Cells were analyzed after 2 days to determine uptake of bromodeoxyuridine (BrdU) (B2506, Sigma), as well as after 12 days to assess the retention of carboxy fluorescein diacetate, succinimidyl ester (BrdU and CFSE assays, see below). To ensure that all neurons and glial cells were newly generated during the cell differentiation period, we analyzed the population of cells derived from spheres 4 hours after attaching and never encountered any cells that expressed neuron-specific beta-III tubulin (TuJ) or GFAP. The neurotrophins BDNF, NT-3, and NGF (R&D Systems) were used in combination with LIF in the experiments shown in Fig. 6.

### Immunocytochemistry

For immunodetection, the cells were fixed with 4% paraformaldehyde in phosphate-buffered saline (PBS, pH7.2) for 15 min at room temperature. Nonspecific binding sites were blocked for 1 hour in 0.1% Triton-100, 1% BSA (wt/vol), and 5% (wt/vol) heat-inactivated goat serum in PBS (PBT1). The fixed cells were incubated overnight at 4°C with diluted antibodies: 1:500 for monoclonal mouse antibody IgG2a to neuron-specific beta-III tubulin (TuJ, MMS-435P; Covance), 1:500 for polyclonal rabbit antibody to GFAP (Dako), 1:500 for polyclonal rabbit antibody to nestin (courtesy of Dr. Ronald McKay, NINDS), and 1:500 for monoclonal mouse antibody to BrdU (B2531, Sigma). Unbound antibodies were removed by three PBT1 washes and one PBT2 (same as PBT1 but without serum) wash for 15 min each at room temperature. FITC-conjugated, TRITC-conjugated, and Cy5-conjugated goat anti-rabbit and anti-mouse secondary antibodies (Jackson ImmunoResearch) were diluted 1:400 in PBS. A 2-hr incubation period in the secondary-antibody mixture preceded three washes for 15 min each in PBS. Counterstaining with short-wavelength nuclear staining agent DAPI (Molecular Probes) was done to visualize cell nuclei. The coverslipped slides were analyzed by fluorescence microscopy and digital image acquisition (Zeiss Axioimager and AxioCam).

### BrdU assay

Equally sized second-generation spheres were plated into gelatin-coated dishes as described above. One batch of spheres was cultured with 5 μg/ml BrdU and 10 ng/ml of LIF in DMEM/high-glucose and F12 media (mixed 1:1) supplemented with N2. The second batch of spheres was cultured with 5 μg/ml BrdU and 0.002% bovine serum albumin (BSA, vehicle control) in the same culture medium. After 48 hours, the differentiated cells were analyzed by immunocytochemistry for uptake of BrdU. This experiment was repeated using 6 different mice, and was compared with a baseline experiment, using the same conditions but stopped after 4 hours.

### CFSE fluorescence

To further assess cell proliferation, we employed carboxyfluorescein diacetate succinimidyl ester (CFSE, Invitrogen, C-1157). CFSE enters the cells by passive diffusion. It is colorless and non-fluorescent until intracellular esterases remove acetate groups from it and convert it to anionic CFSE that fluoresces. Anionic CFSE is well retained within cells and is equally distributed to the daughter cells after mitosis. CFSE labeling has been used as an alternative to standard proliferation analysis techniques such as [^3^H]-thymidine incorporation and BrdU labeling [48]. We used this method because BrdU appeared to be toxic when used for longer than 4 days in our sphere-derived cultures. Spheres were transferred into gelatin-coated dishes and allowed to attach for 3 hours. CFSE was then added to a final concentration of 10 mM and after an incubation period of 18 minutes at 37°C, unincorporated CFSE was washed off with two rinses of PBS. Cells were then cultured at 37°C in medium containing 10 ng/ml of LIF for LIF-treated group and 0.002% BSA for the vehicle control group. The differentiated cells were analyzed by immunocytochemistry after 12 days to assess the retention of CFSE in TuJ-positive and GFAP-positive cells in the LIF-treated group compared to controls. Cy5- and TRITC-conjugated antibodies were used to detect primary antibodies, and DAPI was used to visualize nuclei. The FITC channel was used to visualize CFSE fluorescence. For each experimental data point, we assessed five random areas of at least 30 cells. Settings on the microscope were standardized for each analysis at a fixed gain and an exposure time set at 1500 ms for the FITC channel; background correction was likewise standardized. ImageJ [49] was used for measuring the intensity of CFSE in all TuJ-positive, GFAP-positive cells, and in non-TuJ/non-GFAP-positive cells, and the results were plotted as a histogram.

### Apoptosis assay

Sphere-derived cells, cultured with LIF (10 ng/ml) or without LIF, were analyzed after 48 hours. Recombinant Annexin-V conjugated with FITC (ApoTarget™, Biosource Internatioal, CA USA) was added to the cultures, incubated for 15 minutes, and washed with PBS. The cells were then fixed with 4% PFA and incubated overnight at 4°C with diluted primary antibodies as described above. Cy5-conjugated secondary antibodies were used to detect primary antibodies. DAPI was used to visualize nuclei.

### Statistical analysis

We applied Student's t-test to matched-paired samples and we considered results significant at *p *< 0.05.

### RNA isolation and RT-PCR

Total RNA was isolated using RNeasy Mini kits (Qiagen). Reverse transcription was performed with Superscript III (Invitrogen). The resulting cDNAs were used as templates in polymerase chain reactions using the following primer pairs (forward, reverse, cDNA product length): Otx2 (5'-CCATGACCTATACTCAGGCTTCAGG-3', 5'-GAAGCTCCATATCCCTGGGTGGAAAG-3', 211 bp), islet-1 (5'-CACCTTGCGGACCTGGTATGCC-3', 5'-GCTACCATGCTGTTGGGTGTATC-3', 450 bp), nestin (5'-GCCGAGCTGGAGCGCGAGTTAGAG-3', 5'-GCAAGGGGGAAGAGAAGGATGTCG-3', 694 bp), musashi1 (5'-ACCTACGCCAGCCGGAGTTACAC-3', 5'-CTGGGGCGCTCCTGCTACCTC-3', 444 bp), neurofilament-M (5'-GCACATCACGGTAGAGCGCAAAG-3', 5'-TCGTGCGCGCACTGGAATGCG-3', 450 bp), peripherin (5'-GTGAGCGTAGAGAGCCAGCAGG-3', 5'-TCGAAGCTCTTCCTCCAGCCGT-3', 474 bp), GluR2 (5'-TAAAATGTGGACTTATATGAGGAGTG-3', 5'-CTCTCGATGCCATATACGTTGTAAC-3', 573 bp), GluR3 (5'-GAAAATGTGGTCTTACATGAAATCCG-3', 5'-TGAGTGTTGGTGGCAGGAGCA-3', 525 bp), GluR4 (5'-ATGAGGATTATTTGCAGGCAG-3', 5'-TCAATGAAGGTCTTAGCTGAAG-3', 415 bp), GFAP (5'-CCTCCGCCAAGCCAAACACGAA-3', 5'-ACCATCCCGCATCTCCACAGTC-3', 433 bp), LIFR (5'-CAACCAACAACATGCGAGTG-3', 5'-GGTATTGCCGATGTGTCCTG-3', 680 bp), gp130 (5'-CCACATACGAAGACAGACCA-3', 5'-GCGTTCTCTGACAACACACA-3', 433 bp), NGF (5'-GAAGGAGACTCTGTCCCTGAAGC-3', 5'-TGATGTCTGTGGCTGTGGTCTTA-3', 376 bp), BDNF (5'-CGCAAACATGTCTATGAGGGTTC-3', 5'-TAGTAAGGGCCCGAACATACGAT-3', 302 bp), NT3 (5'-TAGAACCTCACCACGGAGGAAAC-3', 5'-AGGCACACACACAGGAAGTGTCT-3', 359 bp), TrkA(1) (5'-GGTACCAGCTCTCCAACACTGAGG-3', 5'-CCAGAACGTCCAGGTAACTGGGTG-3', 204 bp), TrkA(2) (5'-CAGGGACTAGTGGTGAAGATTGG-3', 5'-TAGCCCAGAACGTCCAGGTAAC-3', 413 bp), TrkB (5'-GTACTGAGCCTTCTCCAGGCATC-3', 5'-CGTCAGGATCAGGTCAGACAAGT-3', 305 bp), TrkC (5'-TACTACAGGGTGGGAGGACACAC-3', 5'-TTTAGGGCAGACTCTGGGTCTCT-3', 225 bp), p75NTR (5'-CCGATGCTCCTATGGCTACTACC-3', 5'-CTATGAGGTCTCGCTCTGGAGGT-3', 353 bp), LIF (5'-CTTACTGCTGCTGGTTCTGCACT-3', 5'-GTAGCATTGAGCTTGACCTGGAG-3', 393 bp), GAPDH (5'-AACGGGAAGCCCATCACCATCTT-3', 5'-CAGCCTTGGCAGCACCAGTGG-3', 442 bp). All RT-PCR results presented were principally confirmed with at least two independent control experiments.

## Competing interests

The author(s) declares that there are no competing interests.

## Authors' contributions

KO and SH conceived of the study. DT, KO, and PS carried out all LIF bioassays. VS conducted the RT-PCR experiments. DT, KO, and SH wrote the manuscript, which has been read and approved by all authors.
